# Design Optimization for a Microfluidic Crossflow Filtration System Incorporating a Micromixer

**DOI:** 10.3390/mi10120836

**Published:** 2019-11-30

**Authors:** Seon Yeop Jung, Jo Eun Park, Tae Gon Kang, Kyung Hyun Ahn

**Affiliations:** 1 School of Chemical and Biological Engineering, Institute of Chemical Processes, Seoul National University, Seoul 08826, Korea; alwaysw8@snu.ac.kr; 2 School of Aerospace and Mechanical Engineering, Korea Aerospace University, Goyang-si, Gyeonggi-do 10540, Korea; p.joeun@gmail.com

**Keywords:** microfluidic filtration, fouling, micromixer, Taguchi method, optimization, numerical simulation

## Abstract

In this study, we report on a numerical study on design optimization for a microfluidic crossflow filtration system incorporated with the staggered herringbone micromixer (SHM). Computational fluid dynamics (CFD) and the Taguchi method were employed to find out an optimal set of design parameters, mitigating fouling in the filtration system. The flow and the mass transfer characteristics in a reference SHM model and a plain rectangular microchannel were numerically investigated in detail. Downwelling flows in the SHM model lead to backtransport of foulants from the permeable wall, which slows down the development of the concentration boundary layer in the filtration system. Four design parameters — the number of grooves, the groove depth, the interspace between two neighboring grooves, and the interspace between half mixing periods — were chosen to construct a set of numerical experiments using an orthogonal array $L_{9}(3^{4})$ from the Taguchi method. The Analysis of Variance (ANOVA) using the evaluated signal-to-noise (SN) ratios enabled us to identify the contribution of each design parameter on the performance. The proposed optimal SHM model indeed showed the lowest growth rate of the wall concentration compared to other SHM models.

## 1. Introduction

Membrane filtration is widely used in a variety of areas, e.g., wastewater treatment, chemical and biological industries, food industry, pharmaceutical industry, and microfluidics [1,2,3,4]. Crossflow filtration (CFF), used for a large-scale application in conventional filtration processes, is a filtration process in which a feed flow is parallel to the membrane surfaces. If the feed flow is directed perpendicular to the membrane surfaces, it is called dead-end filtration. Depending on the pore size, a filtration process is classified into microfiltration, ultrafiltration, and nanofiltration. Two major drawbacks limiting the performance and the reliability of a membrane filtration system are concentration polarization and fouling, leading to flux decline and the decrease in lifetime of membranes [5,6]. Concentration polarization refers to a build-up of the concentration of foulants (molecules or small particles) near the membrane surface, forming a concentration boundary layer. Fouling is an irreversible adsorption of rejected foulants that forms a layer of molecules or particles on the membrane surface.

Typical approaches to enhance flux are the pretreatment of membrane and membrane modifications to obtain fouling-resistant membranes [7,8]. From the viewpoint of fluid mechanics, meanwhile, hydrodynamic techniques such as flow instabilities, inserts, patterned surfaces, and turbulent flow can also be employed to achieve an enhanced performance in crossflow filtration [6,9,10,11,12]. Among such techniques, flow instabilities can be induced by vortical flows, e.g., Taylor vortices in a rotational Couette flow and Dean vortices in a curved channel flow [13,14,15,16]. In the crossflow filtration, such flow instabilities, occurring in coiled or twisted tubular membranes, increase the backtransport of solute or foulants away from the membrane surface, ultimately leading to enhanced permeate fluxes [17,18,19,20]. Another hydrodynamic technique to enhance filtration performance is the use of inserts. Helical baffles inserted in a tubular membranes generate secondary helical flows, suppressing membrane fouling and concentration polarization [21,22].

As far as inserts are concerned, static mixers [23] inducing chaotic advection [24,25] in a laminar flow regime is the most efficient way of utilizing inserts in crossflow filtration [12,26,27]. If chaotic advection takes place in a crossflow filtration system, it leads to chaotic trajectories of fluid particles even in a simple laminar flow condition, preventing suspended particles or solute from adsorbing on the membrane surface. Therefore, mixing via chaotic advection can be regarded as a promising means to mitigate fouling and concentration polarization in a crossflow filtration system working in a laminar flow regime. A recent numerical study conducted by Jung et al. [12] is a representative example, demonstrating the use of a static mixer to suppress the development of the concentration boundary layer and to reduce the wall concentration in a tubular membrane module. In their work, a barrier-embedded partitioned pipe mixer (BPPM) [28,29] is employed as a static mixer inducing chaotic mixing.

With the development of microfabrication technologies in the last decade, microfluidic membrane filtration combining microfluidics and membrane technologies has become a promising research area [30,31,32]. Many different fabrication methods integrating membranes into a microfluidic device have been proposed, e.g., direct incorporation of membranes in microchannels and incorporation of membranes during lithography processes. As for recently developed novel fabrication methods and their applications in microfluidics, we refer to a review paper by Chen and Shen [32]. Since typical flows in microfluidic devices belong to the laminar flow regime, mixing near the membrane surface is not significant and the local concentration of foulants near the membrane surface increases rapidly [32]. Thus, concentration polarization becomes a serious drawback in microfluidic membrane filtration. As in conventional macroscale filtration, membrane fouling is also a factor to be considered when designing a microfluidic filtration system [33,34,35]. In previous studies [36,37], it was demonstrated that, with a micromixer incorporated with a microfluidic membrane filtration system, the permeate flux could be significantly improved via chaotic mixing that reduces the amount of fouling on membrane surfaces. To fully understand the flow kinematics and the mass transport in such filtration systems relying on chaotic mixing, however, further investigation is required. 

To ensure the enhanced performance of a microfluidic filtration system with mixing elements, it is crucial to optimize the mixer geometry and mixing protocols. In this study, we chose the staggered herringbone mixer (SHM) [38] as a micromixer incorporated in a microfluidic crossflow filtration system and demonstrated the design optimization for the filtration system. Our main focus was on optimizing the filtration system using computational fluid dynamics (CFD) and an optimization scheme. In addition, we attempted to elucidate the flow and the mass transfer characteristics in the filtration system with an emphasis on the influence of the unique flow characteristics of the SHM on the development of the wall concentration. To this end, first, the flow and the concentration distribution in the filtration system are investigated using a commercial CFD software, ANSYS-CFX 18.1 (ANSYS Inc., Canonsburg, PA, USA). Then, the Taguchi method [39] is employed to optimize the design variables in such a way that the development of the wall concentration is suppressed. From there, Analysis of Variance (ANOVA) enables us to quantify the influence of each design variable on the development of the wall concentration. Finally, an optimal set of design variables is identified.

## 2. Problem Statement

### 2.1. Geometry of the Crossflow Filtration System

**Figure 1:**
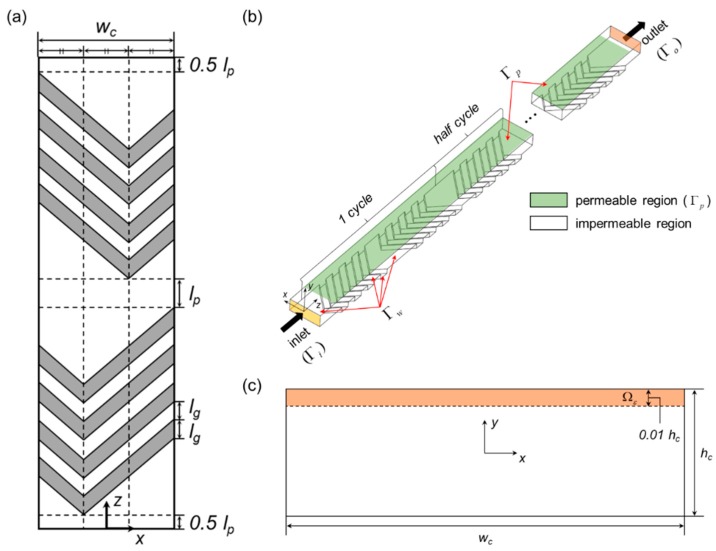
(**a**) Periodic unit of the staggered herringbone mixer (SHM) consisting of four grooves in a half cycle; (**b**) computational domain consisting of 10 periodic units, where a permeable region is located on top of the microchannel, opposite to the herringbone-like grooves; (**c**) a thin rectangular domain ($\Omega_{\varepsilon }$) defined at a cross-section, where the surface-averaged dimensionless wall concentration ${\bar{c}}_{w}$ is evaluated.

Figure 1 illustrates the periodic unit of the staggered herringbone mixer (SHM) with a flat permeable region (membrane) on top of the microchannel, opposite to the bottom with the herringbone-like grooves, for a microfluidic filtration application. The original design suggested by Stroock et al. [38] is referred to determine our SHM designs. As shown in Figure 1a, the location of the apex of a groove is positioned at a distance *1/3 w_c_* from the sidewall in the first half cycle and at a distance *2/3 w_c_* from the sidewall in the last half cycle, where *w_c_* is the channel width. The patterned surface with the grooves creates two counter-rotating cross-sectional flows repeating periodically and inducing chaotic advection [38,40,41]. As the grooves are incorporated in a permeating microchannel, the accumulation of concentration near the permeable wall is expected to be reduced if effective stirring occurs near the membrane surface. Four major design parameters of the SHM — the number of grooves (*n_p_*), the interspace between two neighboring grooves (*l_g_*), the interspace between half mixing periods (*l_p_*), and the groove depth (*h_g_*) — are selected and manipulated, whose specific values are summarized in Section 2.3, to minimize the development of the wall concentration (See Figure 1a for the details). Other geometric parameters are fixed as in [38]; *h_c_* = 77 µm and *w_c_* = 200 µm, where *h_c_* is the channel height. The slanted angle of the grooves is 45°.

Figure 1b shows a typical computational domain, consisting of 10 periodic units of the SHM and two buffer regions near the inlet and outlet. Since a periodic flow field cannot be ensured due to the presence of the permeable region, the computational domain composed of 10 periodic units is used to investigate the flow and the mass transport in the filtration system. The number of spatial periods was determined by referring to previous literatures [40,41], where a significant mixing was identified after ten or so periods. In Section 3.3 and Section 3.4, it is confirmed that the growth rate of the wall concentration in the down-channel direction can be determined from the obtained concentration distributions, demonstrating that the number of periodic units is large enough to capture the development of the wall concentration and the concentration boundary layer. On the other hands, the length of a buffer region is set to be 100 µm, considered to be much larger than the entrance length (*l*_ent_) for the rectangular channel flow (for reference, *l*_ent_ ~ 6 µm, when Re = 1 [42]). Two buffer regions are inserted to avoid any numerical artifact caused by the presence of abrupt permeate flux near the inlet and outlet of the channel. The origin of the coordinate is located at the center of the inlet. A thin rectangular domain ($\Omega_{\varepsilon }$) defined at a cross-section, shown in Figure 1c, will be used to evaluate the surface-averaged dimensionless wall concentration ${\bar{c}}_{w}$ as defined in Equation (4) in Section 2.4.

### 2.2. Governing Equations and Boundary Conditions

The objective function in the design optimization of the microfluidic filtration system is to minimize the development of the wall concentration on the permeable region. Therefore, the evolution of the wall concentration in the down-channel direction should be obtained. To find the concentration distribution affected by the flow in a specific channel design, we solve the flow and mass transport problems in a decoupled manner. A steady flow of an incompressible Newtonian fluid in a laminar flow regime is assumed. The velocity field is obtained by solving the steady Navier-Stokes equation and the continuity equation, given by

(1) $$\rho \left(u\cdot \nabla u\right)=-\nabla p+\mu \nabla^{2}u\;\mathrm{in}\;\Omega$$

(2) ∇⋅u=0    in Ω

where ρ is the density, u the velocity, μ the viscosity, and p the pressure. A uniform inlet velocity $\bar{u}$ is imposed at the inlet ($\Gamma_{i}$). The zero pressure boundary condition (p=0) is imposed at the outlet ($\Gamma_{o}$). At the permeable region ($\Gamma_{p}$), a uniform normal permeate velocity is imposed as a Dirichlet boundary condition, given by $u\cdot n=u_{per}$, where $u_{per}$ is the magnitude of the permeate velocity and n the outward unit normal vector at the boundary. In this study, $u_{per}$ is fixed to $u_{per}=1\times 10^{-4}\bar{u}$, which lies in a typical operational condition of a microfiltration application. This assumption of a constant permeate velocity holds when the applied transmembrane pressure ($\Delta p_{m}$) is much higher than the transmembrane osmotic pressure ($\Delta \pi_{m}$), which is frequently assumed in previous studies [43,44]. At the remaining boundaries (denoted by $\Gamma_{w}$) — corresponding to the bottom wall, side walls, surface of the grooves, and buffer region — the no-slip boundary condition (u=0) is imposed. The Reynolds number (Re) is defined as Re=ρu¯Dh/μ, where $D_{h}$ is the hydraulic diameter ($D_{h}=w_{c}h_{c}/2(w_{c}+h_{c})$). It is assumed that Re=1 in our numerical simulation, since we are concerned with filtration in a microfluidic device.

The concentration distribution of foulants is obtained by solving a steady convection-diffusion equation using the velocity field obtained as a solution of the flow problem. Although complex fouling behaviors would exist in real applications, due to shear-induced migration, frictional dynamics, cell adhesion, and diffusiophoresis, we focus on the simplest mass transfer driven by convection and diffusion to investigate the fouling mitigation by chaotic mixing. The steady convection-diffusion equation is given by

(3) ∇⋅(𝒟∇c)−u⋅∇c=0    in Ω

where *𝒟* is the diffusivity of the foulant and c the concentration of the foulant. A uniform concentration ($c=c_{0}$, where $c_{0}$ is a constant) is imposed at the inlet ($\Gamma_{i}$), which is the case when a well-dispersed feed solution is introduced through the inlet. At the outlet ($\Gamma_{o}$), the diffusive mass transport is zero, i.e., n⋅𝒟∇c=0. At the remaining boundaries ($\Gamma_{p}$ and $\Gamma_{w}$), the total mass flux is zero, i.e., n⋅(−𝒟∇c+cu)=0. It should be noted that the zero flux condition at $\Gamma_{p}$ corresponds to the case of 100% rejection of the foulant by the permeable membrane. The Péclet number (Pe) is defined as $\mathrm{Pe}=\bar{u}D_{h}/𝒟$, fixed at $\mathrm{Pe}=10^{7}$ in this study, corresponding to the case where the transport of foulants is dominated by the convective transport. For other details on the problem statement, we refer to our previous study [12].

### 2.3. Design Parameters

For the optimal design of SHM minimizing the degree of fouling (which is characterized by the rate of increase of the wall concentration), the Taguchi method is employed to conduct the optimization and sensitivity analysis. Table 1 summarizes the levels of four selected design parameters. We adopted the SHM design proposed in the previous studies [38,45] to determine the range of variation for each parameter. We choose a reference SHM model that is constructed with the design parameters, $n_{p}=4$, $l_{g}=56.6\;\mu m$, $l_{p}=56.6\;\mu m$, and $h_{g}=12\;\mu m$. Our numerical analysis is carried out for nine SHM models derived from the $L_{9}(3^{4})$ orthogonal array of the Taguchi method. Since the three parameters ($n_{p}$, $l_{g}$, and $l_{p}$) are related to the length of one periodic unit of a specific SHM design, the length of a simulation domain with ten SHM units varies with the combination of the design parameters. 

### 2.4. Characterization of the Degree of Fouling

To assess the degree of fouling in a SHM model, one needs to define the wall concentration on the permeable region. As used in our previous study [12], a surface-averaged dimensionless wall concentration (${\bar{c}}_{w}$) is defined to avoid a possible numerical artifact in the concentration values at wall nodes, given by:

(4) $${\bar{c}}_{w}=\frac{\int_{\Omega_{\varepsilon }}c\left(x\right)dA}{\int_{\Omega_{\varepsilon }}c_{0}dA}$$

where $\Omega_{\varepsilon }$ is a thin rectangular domain defined in a cross-section of interest at a specific *z* coordinate (See Figure 1c) and x the position vector for a location in the cross-section. In optimization, we will use an objective function, the minimization of the growth rate of the dimensionless wall concentration in the down-channel direction, rather than minimizing the wall concentration at a fixed location. The objective function is an appropriate one since the channel length varies with the design parameters of the SHM. The growth rate of the wall concentration is obtained from the regression of the average wall concentration ${\bar{c}}_{w}$ as a function of the axial coordinate z. 

To characterize the growth of the wall concentration ${\bar{c}}_{w}$, we adopt the film theory developed for a thin slit channel [46,47]. In this theory, the dimensionless concentration difference is represented by

(5) $$\frac{c_{w}-c_{p}}{c_{b}-c_{p}}=\exp\left(\frac{u_{per}\delta }{𝒟}\right)$$

where $c_{w}$ is the wall concentration, $c_{b}$ the bulk concentration, $c_{p}$ the permeate concentration, $u_{per}$ the permeate velocity, and δ the concentration boundary layer thickness. From the film theory for a fully developed laminar flow in the slit channel, the concentration boundary layer thickness (δ) is represented by

(6) $$\frac{\delta \left(z\right)}{z}=1.475\;{\left(\frac{h}{z}\right)}^{2/3}{\left(\frac{𝒟}{u_{\max}h}\right)}^{1/3}$$

where h is the channel height and $u_{\max}$ the maximum fluid velocity in the fully developed laminar flow. Since we assume 100% rejection of foulants on the permeable wall, the permeate concentration is zero, i.e., $c_{p}=0$. Rearranging Equations (5) and (6), the dimensionless wall concentration becomes

(7) $$\ln\left(\frac{c_{w}}{c_{b}}\right)=1.475{\left(\frac{𝒟}{u_{\max}h}\right)}^{1/3}\;{\left(\frac{h^{2}u_{per}{}^{3}}{{𝒟}^{3}}z\right)}^{1/3}$$

Motivated by Equation (7), we propose to use an exponential form to describe the change of the dimensionless wall concentration ${\bar{c}}_{w}$ with respect to $\hat{z}$, given by

(8) $${\bar{c}}_{w}=\exp\left(a{\hat{z}}^{n}\right)$$

where n is an exponent representing the growth rate of the fouling layer along the down-channel direction and $\hat{z}=z/w_{c}$. The two parameters (a and n) in Equation (8) are determined by curve fitting.

### 2.5. Numerical Methods

Numerical simulations are performed using a commercial CFD software, ANSYS-CFX 18.1 (ANSYS, Inc.). A simulation domain is discretized by hexahedral elements. The number of nodes ranges from 12,180,401 to 23,699,911, while the number of elements from 11,775,360 to 22,910,400, for channel geometries used in this study. Since more than one million elements are generated in one periodic unit of a SHM model, it is considered to be fine enough to avoid grid dependency, referring to the previous literatures [40,41], where less than one million elements are used in mixing analysis to reproduce experimentally observed mixing patterns. Refined boundary-layer elements with the minimum size being 0.25 μm (equivalent to 0.003 *h_c_*) are generated near the permeable wall to obtain reliable concentration values. A workstation with two 10-core processors (Intel(R) Xeon(R) CPUs E5-2687 W 3.1 GHz) and 512 GB of memory is used for our numerical simulation.

## 3. Results and Discussion

### 3.1. Development of the Concentration Boundary Layer in a Plain Rectangular Channel

Before conducting numerical simulations for the SHM models, we first solve the flow and mass transfer problems in a plain rectangular channel (without any mixing element) to investigate the development of the wall concentration and the concentration boundary layer. A plain rectangular channel with the same cross-sectional dimensions as those of the SHM (*h_c_* = 77 µm and *w_c_* = 200 µm) is used in the simulations. The same boundary conditions and material properties as used for the SHM models are used to solve the flow and mass transport problem for the plain rectangular channel.

**Figure 2:**
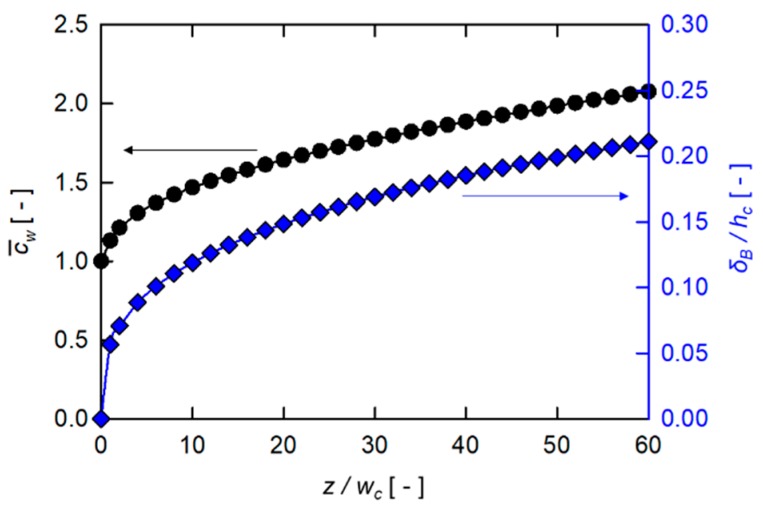
The surface-averaged dimensionless wall concentration (${\bar{c}}_{w}$) and the dimensionless concentration boundary layer thickness ($\delta_{B}/h_{c}$ ) in the plain rectangular channel as functions of the dimensionless axial coordinate, ${z/w}_{c}$.

Figure 2 illustrates the evolution of the surface-averaged dimensionless wall concentration (${\bar{c}}_{w}$) and the dimensionless concentration boundary layer thickness ($\delta_{B}/h_{c}$) in the down-channel direction of the plain rectangular channel. The concentration boundary layer thickness ($\delta_{B}$) is defined by a criterion, given by

(9) $${\frac{c^{*}-{\bar{c}}_{w}}{c_{b}^{*}-{\bar{c}}_{w}}|}_{y=\delta_{B}}=0.01,$$

where $c^{*}=c/c_{0}$ and $c_{b}^{*}=c_{b}/c_{0}$ ($c_{b}^{*}=1$, in this study), which is analogous to that used to define the thermal boundary layer in heat transfer [42]. As expected by Equations (6) and (7) derived from the film theory, the dimensionless wall concentration (${\bar{c}}_{w}$) and the dimensionless boundary layer thickness ($\delta_{B}/h_{c}$) keep increasing with ${z/w}_{c}$. Since ${\bar{c}}_{w}$ is defined as the surface-averaged wall concentration scaled by the inlet concentration, it starts from the initial value of 1. It should be noted here that $\delta_{B}/h_{c}\propto z^{0.325}$, i.e., the power-law index n for the plain channel is 0.325, while that predicted from the film theory is 1/3, thus the relative difference of the two values is less than 3%.

### 3.2. Flow Characteristics of the Shm with a Permeable Wall 

**Figure 3:**
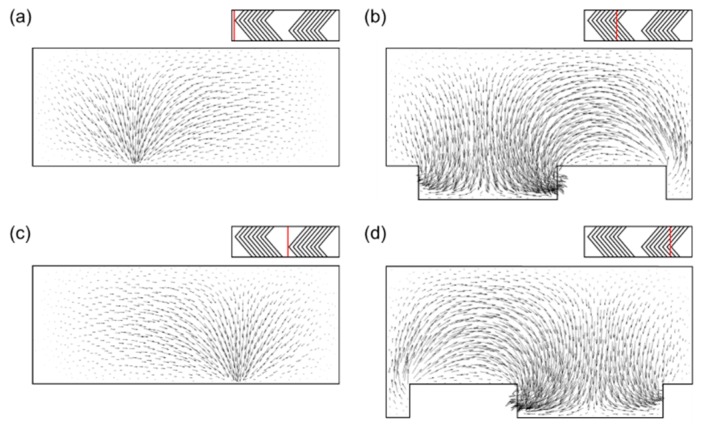
The velocity vector plots at four cross sections showing two counter-rotating flow portraits and downwelling flows in the reference SHM model. Velocity vectors are projected on the cross-sections. The axial location of each cross section is indicated by a red line. Velocity vectors (**a**) at the first apex in the first half cycle; (**b**) at the last apex in the first half cycle; (**c**) at the first apex in the second half cycle; and (**d**) at the last apex in the second half cycle.

In the SHM, two helical flows are generated in each half cycle by the herringbone-like grooves inducing lateral motions of the fluid [38,40]. Due to the apex location changing alternatingly in a periodic unit, two cross-sectional flow portraits are generated in the first and second half cycles and they intersect one another when projected onto the same plane. According to the linked twisted maps (LTM) framework [48], the two flow portraits satisfy the necessary condition for the creation of chaotic advection [49,50]. Figure 3 depicts cross-sectional velocity vectors projected onto planes normal to the z direction. The red lines indicate the axial positions used to plot cross-sectional velocity vectors. One can clearly observe that downwelling flows are generated when a fluid stream passes through the apex of a herringbone-like groove, leading to two counter-rotating cross-sectional flows [40]. Due to asymmetric groove patterns repeating periodically, the location of the downwelling flow changes periodically as well, which is the unique flow characteristic of the SHM. It is worth mentioning that, in spite of the outflux at top surface, the cross-sectional flow pattern is similar to that observed in the SHM with non-permeable walls, which is due to the small permeation velocity compared to the average inlet velocity as mentioned in Section 2.2. The two counter-rotating flows repeating periodically are able to increase the backtransport of foulants from the permeable wall, effectively mitigating concentration polarization and membrane fouling in the microfluidic crossflow filtration. The concentration distribution of foulants affected by the flow in the filtration system with the SHM will be discussed in the following section.

### 3.3. Concentration Distribution of Foulants

**Figure 4:**
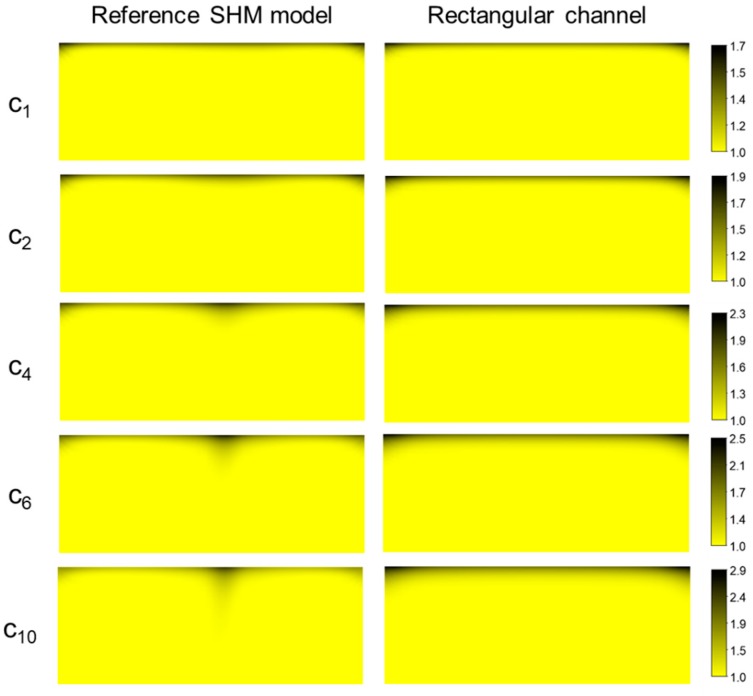
Evolution of the concentration distribution in the reference SHM model and the plain rectangular channel with the permeating wall (upper wall). The color contour represents the dimensionless concentration ($c/c_{0}$), with black region having a higher accumulation of foulants. Here, subscripts indicate the spatial periods of the SHM model in which the concentration contours are plotted.

Figure 4 illustrates the cross-sectional concentration distributions in the reference SHM model (where $n_{p}=4$, $l_{g}=56.6\;\mu m$, $l_{p}=56.6\;\mu m$, and $h_{g}=12\;\mu m$) and in the plain rectangular channel, both with the constant permeation velocity $u_{per}$ on $\Gamma_{p}$, demonstrating the development of the concentration boundary layer near the permeable region. As for the reference SHM model, the concentration distribution at the end of the five selected periods are shown, where each subscript indicates the corresponding spatial period (i.e., C_2_ indicates the concentration at the end of the second period, and so on). In the case of the plain rectangular channel, the concentration distributions are plotted at the same cross-sections used in the reference SHM model. Due to the downwelling flows present in the SHM model (See Figure 3), foulants accumulated near the upper wall (permeable wall) were dragged downward, thus slowing down the concentration boundary layer development. Compared to the concentration distribution in the plain rectangular channel, the boundary layer in the SHM model was thinner, especially around $x=\pm 0.25w_{c}$, clearly demonstrating that the accumulated mass was back-transported toward the bulk region by the rotational motions of the fluid. However, the wall concentration near the center (x=0) was locally high due to the two counter-rotating flow patterns merged at the center. It is apparent that, even though the SHM model is not an optimized one, the flow and mixing characteristics in the SHM lead to the reduction of the wall concentration in an overall sense, compared to the case of the plain rectangular channel.

**Figure 5:**
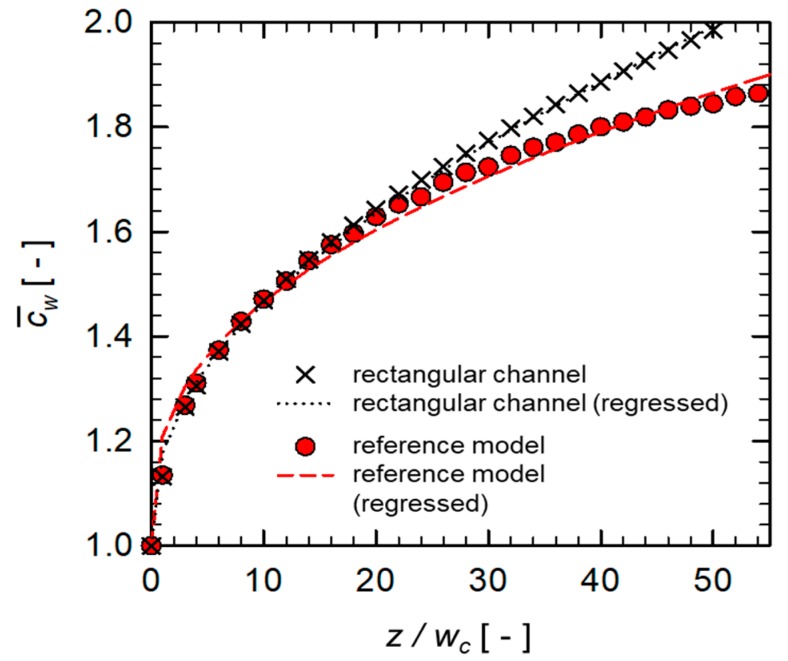
Evolution of ${\bar{c}}_{w}$ in the reference SHM model and in the plain rectangular channel along the down-channel direction. Regression lines are obtained with the function defined by Equation (8).

Figure 5 shows the evolution of ${\bar{c}}_{w}$ in the reference SHM model and in the plain rectangular channel along the down-channel direction. It was observed that the growth of ${\bar{c}}_{w}$ was suppressed by introducing the SHM in the channel, compared to that in the plain rectangular channel. As qualitatively observed in Figure 4, the downwelling flows induced by the herringbone-like grooves lead to the slowdown of the growth rate of the wall concentration ${\bar{c}}_{w}$. To quantify the growth rate of ${\bar{c}}_{w}$, we performed a linear regression to find the exponent *n* in the model equation, Equation (8), and plotted the regression lines using the fitted parameters (a and n) for the two cases (See Figure 5). The regression line of the SHM model slightly deviated from the numerically obtained data points. The deviation is thought to be caused by adopting the film theory, which was originally developed for an infinitely thin slit channel without considering any geometric feature in the channel, which is not the case for the SHM model. In the case of the plain rectangular channel, however, the regression line matched well with the data points. The fitted exponent n in the plain rectangular channel was 0.368, which is higher than that predicted from the film theory given by Equation (7). Since we are concerned with the plain rectangular channel with a finite width, there is a possibility of a deviation from the predicted value using the film theory in which an infinite channel width is assumed. 

### 3.4. Design Optimization using the Taguchi Method

**Figure 6:**
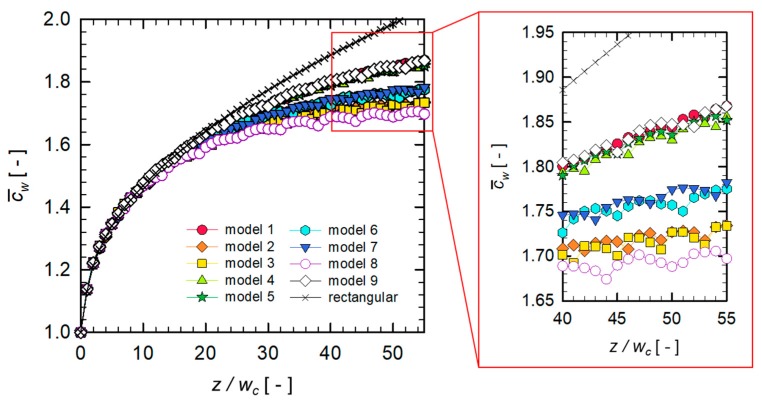
Evolution of ${\bar{c}}_{w}$ in nine SHM models constructed by the $L_{9}(3^{4})$ orthogonal array along the down-channel direction. The reference SHM model corresponds to the model 1.

In the previous section, we learned that the flow in the SHM can reduce the growth rate of the wall concentration in the down-channel direction. Now, we attempt to optimize the microfluidic filtration system, the SHM with a permeable wall. The objective function is to minimize the growth exponent n of the dimensionless wall concentration ${\bar{c}}_{w}$. As described in Section 2.3, we construct nine models (for numerical experiments) with a specific combination of design parameters provided by the $L_{9}(3^{4})$ orthogonal array. For each SHM model, the flow and mass transfer problems were solved and a linear regression was performed to find the exponent n. Figure 6 shows the evolution of ${\bar{c}}_{w}$ in each SHM model along the down-channel direction. In all SHM designs, a remarkable reduction of ${\bar{c}}_{w}$ was observed, compared to that in the plain rectangular channel. In this figure, a periodic fluctuation in ${\bar{c}}_{w}$ is observed in each SHM model, because the concentration distribution is affected by the periodically rotating flow nature. Table 2 summarizes the orthogonal array and the fitted exponent n in each SHM model, which will be used to evaluate the signal-to-noise (SN) ratio for the model.

**Figure 7:**
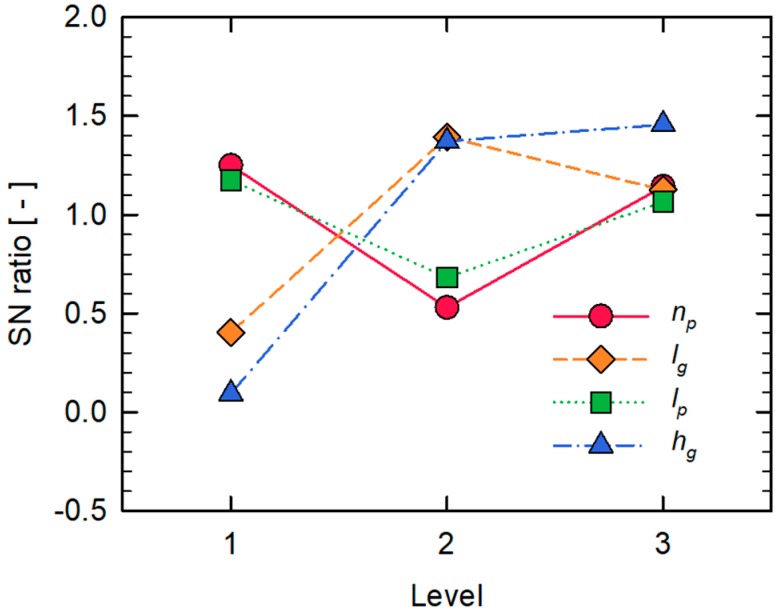
SN ratios of the four design parameters with three levels.

**Figure 8:**
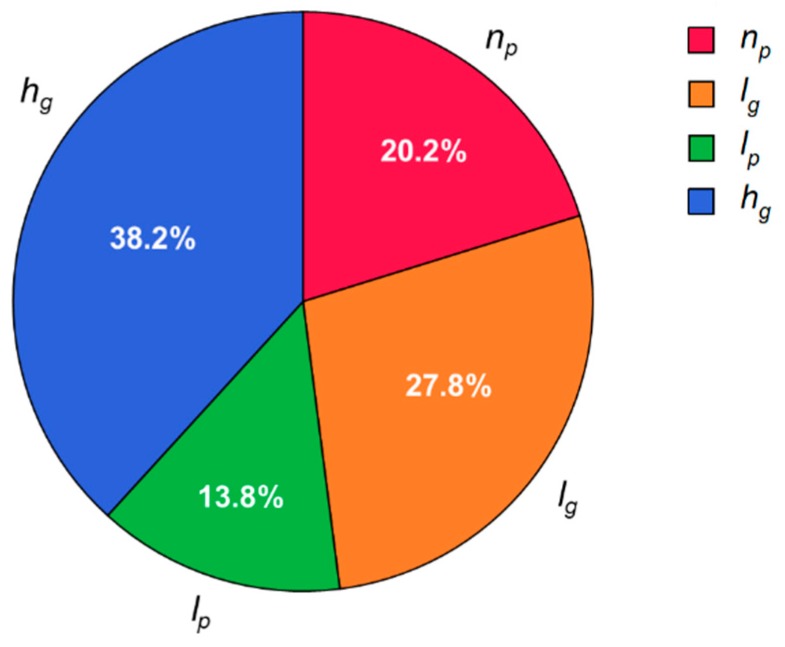
Contribution of each parameter on the performance.

Since a lower value of n is desirable to mitigate fouling, the performance analysis was carried out with the lower-the-better type [39], where the SN ratio is defined by

(10) $$\mathrm{SN}\;\mathrm{ratio}=-10\log_{10}{\left(\frac{n}{n_{0}}\right)}^{2}$$

where $n_{0}$ is the growth exponent of the reference SHM model. By investigating the SN ratio, an optimal combination of design parameters of the SHM model that are expected to minimize the growth exponent n would be determined within the limit of the selected levels of the design parameters. The contribution of each design parameter on the performance can be estimated from the Analysis of Variance (ANOVA). Figure 7 shows the SN ratios of different levels in each design parameter. In this plot, the optimal set of design parameters can be obtained by selecting the level with the highest SN ratio for each design parameter. The contribution of each design parameter on the growth exponent, identified by the ANOVA, is shown in Figure 8. It shows that the groove depth $h_{g}$ is the most significant parameter, while the interspace between two half cycles $l_{p}$ shows the weakest contribution on the performance. As for $h_{g}$, it was reported in previous studies [40,45] that the groove depth is highly related to the strength of the rotational flows induced in the SHM, thus also to the strength of the backtransport of foulants caused by the downwelling flows. 

**Figure 9:**
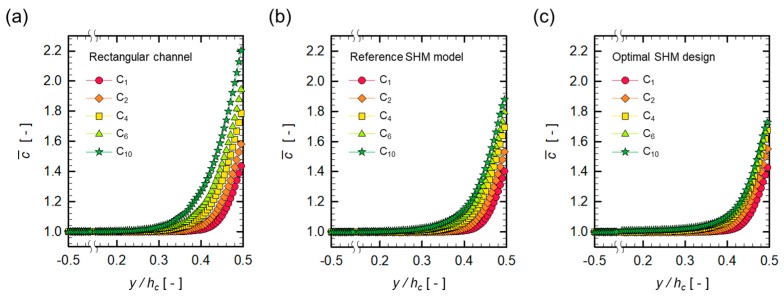
Evolution of the concentration profile at five spatial periods; (**a**) the plain rectangular channel; (**b**) the reference SHM model; and (**c**) the optimal SHM design. The subscripts in the legend indicate the spatial periods in which the concentration profiles are evaluated.

Table 3 summarizes the optimal combination of design parameters and the corresponding growth exponent obtained from the concentration distributions for the optimal SHM design. It should be noted that the optimal SHM design leads to the smallest value of n less than that of any SHM model (See Table 2), showing the best performance with regard to minimizing the growth rate of the dimensionless wall concentration ${\bar{c}}_{w}$. Figure 9 illustrates the evolution of the concentration in the vertical direction (in the y-direction) at several periods for three channel designs, the plain rectangular channel, the reference SHM model, and the optimized SHM model. The ordinate in the figure is the surface-averaged dimensionless concentration $\bar{c}$ at a vertical location y, defined by

(11) $$\bar{c}=\frac{\int_{\Omega_{y}}c\left(x\right)dA}{\int_{\Omega_{y}}c_{0}dA}$$

where $\Omega_{y}$ is a thin rectangular slab with the height $0.01\;h_{c}$ and the width $w_{c}$, defined at a cross-section and centered at a specific y coordinate. In all cases, the wall concentration increases as the number of the spatial period increases, but with a different growth rate. The growth rate of $\bar{c}$ decreases significantly by the introduction of the SHM, compared to that observed in the plain rectangular channel. A further reduction in the growth rate is achieved with the optimal SHM design. 

## 4. Conclusions

In this study, design optimization for the staggered herringbone mixer (SHM) incorporated in a microfluidic crossflow filtration system was carried out using the Taguchi method and computational fluid dynamics (CFD) and the Taguchi method. Four design parameters of the SHM were chosen and nine sets of SHM models with different combinations of the design parameters were constructed by the $L_{9}(3^{4})$ orthogonal array. Numerical simulations were conducted using a commercial CFD software, ANSYS-CFX 18.1 (ANSYS Inc.), to obtain the flow and mass transfer characteristics for each model. Downwelling flows observed in the reference SHM model induced the back-transport of accumulated mass away from the permeable region, ultimately reducing the wall concentration (${\bar{c}}_{w}$). The growth exponent n of ${\bar{c}}_{w}$ along the down-channel direction was determined by the linear regression applied to a set of data for ${\bar{c}}_{w}$ and $z/w_{c}$. The signal-to-noise (SN) ratio for each design parameter with three different levels enabled us to identify an optimal SHM design and the Analysis of Variance (ANOVA) was used to quantify the contribution of each design parameter on the performance. The optimized SHM design resulted in the lowest value of the growth rate of ${\bar{c}}_{w}$ and the most suppressed concentration distribution near to the permeable region, compared to other SHM models. To the best of our knowledge, this research is the first attempt to optimize a microfluidic filtration system to mitigate the development of fouling (in this study, characterized by the growth exponent n). As far as the optimization scheme is concerned, our methodology was not a sophisticated one, but it indeed enabled us to find key parameters with influence on the filtration performance and a set of optimal design parameters.

## Figures and Tables

**Table 1:** Design parameters of the SHM and their range of variation.

Levels	Parameters			
	$n_{p}$	$l_{g}{}^{*}$	$l_{p}{}^{*}$	$h_{g}{}^{*}$
1	4	56.6	56.6	12
2	6	70.7	70.7	17
3	8	84.8	84.8	22

^*^ The unit of length is μm.

**Table 2:** The $L_{9}(3^{4})$ orthogonal array and the growth exponent (n ) of ${\bar{c}}_{w}$ in each SHM model.

Model	Parameter Levels				n
	$n_{p}$	$l_{g}$	$l_{p}$	$h_{g}$	
1 (reference)	1	1	1	1	0.304
2	1	2	2	2	0.247
3	1	3	3	3	0.242
4	2	1	2	3	0.298
5	2	2	3	1	0.298
6	2	3	1	2	0.262
7	3	1	3	2	0.269
8	3	2	1	3	0.234
9	3	3	2	1	0.299

**Table 3:** The optimal design of the SHM to minimize the growth exponent (n) of ${\bar{c}}_{w}$.

$n_{p}$	$l_{g}$	$l_{p}$	$h_{g}$	n
1	2	1	3	0.225

## References

[1] Belfort G., Davis R.H., Zydney A.L. (1994). The behavior of suspensions and macromolecular solutions in crossflow microfiltration. J. Membr. Sci..

[2] de Jong J., Lammertink R.G.H., Wessling M. (2006). Membranes and microfluidics: A review. Lab Chip.

[3] Agboola O. (2019). The role of membrane technology in acid mine water treatment: A review. Korea. J. Chem. Eng..

[4] Park J., Willenbacher N., Ahn K.H. (2019). How the interaction between styrene-butadiene-rubber (SBR) binder and a secondary fluid affects the rheology, microstructure and adhesive properties of capillary-suspension-type graphite slurries used for Li-ion battery anodes. Colloids Surf. A. Physciochem. Eng. Asp..

[5] van der Bruggen B., Mänttäri M., Nyström M. (2008). Drawbacks of applying nanofiltration and how to avoid them: A review. Sep. Purif. Technol..

[6] Jaffrin M.Y. (2012). Hydrodynamic Techniques to Enhance Membrane Filtration. Annu. Rev. Fluid Mech..

[7] Gao W., Liang H., Ma J., Han M., Chen J.-L., Han Z.-S., Li G.-B. (2011). Membrane fouling control in ultrafiltration technology for drinking water production: A review. Desalination.

[8] Kang G.-D., Cao Y.-M. (2012). Development of antifouling reverse osmosis membranes for water treatment: A review. Water Res..

[9] Winzeler H.B., Belfort G. (1993). Enhanced performance for pressure-driven membrane processes: The argument for fluid instabilities. J. Membr. Sci..

[10] Jung S.Y., Won Y.-J., Jang J.H., Yoo J.H., Ahn K.H., Lee C.-H. (2015). Particle deposition on the patterned membrane surface: Simulation and experiments. Desalination.

[11] Jung S.Y., Ahn K.H. (2019). Transport and deposition of colloidal particles on a patterned membrane surface: Effect of cross-flow velocity and the size ratio of particle to surface pattern. J. Membr. Sci..

[12] Jung S.Y., Jung H.I., Kang T.G., Ahn K.H. (2019). Fouling mitigation in crossflow filtration using chaotic advection: A numerical study. AIChE J..

[13] Dutta P.K., Ray A.K. (2004). Experimental investigation of Taylor vortex photocatalytic reactor for water purification. Chem. Eng. Sci..

[14] Moll R., Veyret D., Charbit F., Moulin P. (2007). Dean vortices applied to membrane process. Part II: Numerical approach. J. Membr. Sci..

[15] Ock J.H., Hong J.S., Ahn K.H. (2018). Acceleration of instability during the capillary thinning process due to the addition of particles to a poly(ethylene oxide) solution. J. Non-Newton. Fluid Mech..

[16] Jin H., Kang K., Ahn K.H., Briels W.J., Dhont J.K.G. (2018). Non-local stresses in highly non-uniformly flowing suspensions: the shear-curvature viscosity. J. Chem. Phys..

[17] Mallubhotla H., Belfort G. (1997). Flux enhancement during Dean vortex microfiltration. 8. Further diagnostics. J. Membr. Sci..

[18] Moulin P., Manno P., Rouch J.C., Serra C., Clifton M.J., Aptel P. (1999). Flux improvement by Dean vortices: ultrafiltration of colloidal suspensions and macromolecular solutions. J. Membr. Sci..

[19] Ghogomu J.N., Guigui C., Rouch J.C., Clifton M.J., Aptel P. (2001). Hollow-fibre membrane module design: comparison of different curved geometries with Dean vortices. J. Membr. Sci..

[20] Norouzi M., Sedaghat M.H., Shahmardan M.M., Nobari M.R.H. (2015). On the origin of viscoelastic Taylor–Couette instability resulted from normal stress differences. Korea-Aust. Rheol. J..

[21] Gupta B.B., Howell J.A., Wu D., Field R.W. (1995). A helical baffle for cross-flow microfiltration. J. Membr. Sci..

[22] Akagi T., Horie T., Masuda H., Matsuda K., Matsumoto H., Ohmura N., Hirata Y. (2018). Improvement of separation performance by fluid motion in the membrane module with a helical baffle. Sep. Purif. Technol..

[23] Ghanem A., Lemenand T., Valle D.D., Peerhossaini H. (2014). Static mixers: mechanisms, applications, and characterization methods — A review. Chem. Eng. Res. Des..

[24] Aref H. (1984). Stirring by chaotic advection. J. Fluid Mech..

[25] Ottino J.M. (1990). Mixing, chaotic advection, and turbulence. Annu. Rev. Fluid Mech..

[26] Armbruster S., Cheong D., Lölsberg J., Popovic S., Yüce S., Wessling S. (2018). Fouling mitigation in tubular membranes by 3D-printed turbulence promoters. J. Membr. Sci..

[27] Armbruster S., Stockmeier F., Junker M., Schiller-Becerra M., Yüce S., Wessling M. (2020). Short and spaced twisted tapes to mitigate fouling in tubular membranes. J. Membr. Sci..

[28] Jung S.Y., Ahn K.H., Kang T.G., Park G.T., Kim S.U. (2018). Chaotic mixing in a barrier-embedded partitioned pipe mixer. AIChE J..

[29] Jung H.I., Jung S.Y., Kang T.G., Ahn K.H. (2018). Numerical study on the mixing in a barrier-embedded partitioned pipe mixer (BPPM) for non-creeping flow conditions. Korea-Aust. Rheol. J..

[30] Ngene I.S., Lammertink R.G.H., Wessling M., van der Meer W. (2010). A microfluidic membrane chip for in situ fouling characterization. J. Membr. Sci..

[31] Chen X., Shen J., Hu Z., Huo X. (2016). Manufacturing methods and applications of membranes in microfluidics. Biomed. Microdevices.

[32] Chen X., Shen J. (2017). Review of membranes in microfluidics. J. Chem. Technol. Biotechnol..

[33] Warkiani M.E., Wicaksana F., Fane A.G., Gong H.-Q. (2015). Investigation of membrane fouling at the microscale using isopore filters. Microfluid. Nanofluid..

[34] Linkhorst J., Beckmann T., Go D., Kuehne A.J.C., Wessling M. (2016). Microfluidic colloid filtration. Sci. Rep..

[35] Completo C., Geraldes V., Semião V., Mateus M., Rodrigues M. (2019). Comparison between microfluidic tangential flow nanofiltration and centrifugal nanofiltration for the concentration of small-volume samples. J. Membr. Sci..

[36] Ho C.K., Altman S.J., Jones H.D.T., Khalsa S.S., McGrath L.K., Clem P.G. (2008). Analysis of micromixers to reduce biofouling on reverse-osmosis membranes. AIChE J..

[37] Himstedt H.H., Yang Q., Dasi L.P., Qian X., Wickramasinghe S.R., Ulbricht M. (2011). Magnetically Activated Micromixers for Separation Membranes. Langmuir.

[38] Stroock A.D., Dertinger S.K.W., Ajdari A., Mezić I., Stone H.A., Whitesides G.M. (2002). Chaotic Mixer for Microchannels. Science.

[39] Roy R.K. (1990). A Primer on the Taguchi Method.

[40] Kang T.G., Kwon T.H. (2004). Colored particle tracking method for mixing analysis of chaotic micromixers. J. Micromech. Microeng..

[41] Kang T.G., Singh M.K., Kwon T.H., Anderson P.D. (2008). Chaotic mixing using periodic and aperiodic sequences of mixing protocols in a micromixer. Microfluid. Nanofluid..

[42] Bergman T.L., Lavine A.S., Incropera F.P., DeWitt D.P. (2017). Fundamentals of Heat and Mass Transfer.

[43] Oxarango L., Schmitz P., Quintard M. (2004). Laminar flow in channels with wall suction or injection: A new model to study multi-channel filtration systems. Chem. Eng. Sci..

[44] Venezuela A.L., Pérez-Guerrero J.S., Fontes S.R. (2009). Hybrid modeling of convective laminar flow in a permeable tube associated with the cross-flow process. Commun. Nonlinear Sci..

[45] Ansari M.A., Kim K.Y. (2007). Shape optimization of a micromixer with staggered herringbone groove. Chem. Eng. Sci..

[46] Porter M.C. (1972). Concentration polarization with membrane ultrafiltration. Ind. Eng. Chem. Prod. Res. Develop..

[47] Kim S., Hoek E.M.V. (2005). Modeling concentration polarization in reverse osmosis processes. Desalination.

[48] Wiggins S., Ottino J.M. (2004). Foundations of chaotic mixing. Phil. Trans. R. Soc. Lond. A.

[49] Kang T.G., Hulsen M.A., Anderson P.D., den Toonder J.M.J., Meijer H.E.H. (2007). Chaotic mixing induced by a magnetic chain in a rotating magnetic field. Phys. Rev. E..

[50] Kang T.G., Anderson P.D. (2014). The effect of inertia on the flow and mixing characteristics of a chaotic serpentine mixer. Micromachines.

